# Malignancy in Ground-Glass Opacity Using Multivariate Regression and Deep Learning Models: A Proof-of-Concept Study

**DOI:** 10.3390/jcm14228082

**Published:** 2025-11-14

**Authors:** Abed Agbarya, Edmond Sabo, Mohammad Sheikh-Ahmad, Leonard Saiegh, Mor Pincas, Miguel Gorenberg, Walid Shalata, Dan Levy Faber

**Affiliations:** 1 Department of Oncology, Bnai-Zion Medical Center, 47 Eliyahu Golomb Avenue, Haifa 3339419, Israel; 2 The Ruth and Bruce Rappaport Faculty of Medicine, Technion, Israel Institute of Technology, 1 Efron Street, Haifa 3339101, Israel; mohammad.ahmad@b-zion.org.il (M.S.-A.); mor.pincas@campus.technion.ac.il (M.P.); miguel.gorenberg@b-zion.org.il (M.G.); danfaber1@hotmail.com (D.L.F.); 3 Department of Pathology, Carmel Medical Center, 7 Michal Street, Haifa 3436212, Israel; edmondsa@clalit.org; 4 Institute of Endocrinology, Bnai-Zion Medical Center, 47 Eliyahu Golomb Avenue, Haifa 3339419, Israel; leonard.saiegh@gmail.com; 5 Department of Nuclear Medicine, Bnai Zion Medical Center, 47 Eliyahu Golomb Avenue, Haifa 3339419, Israel; 6 The Legacy Heritage Cancer Center, Dr. Larry Norton Institute, Soroka Medical Center, Beer Sheva 8410501, Israel; walid.shalata@gmail.com; 7 Faculty of Health Sciences, Ben Gurion University of the Negev, Beer Sheva 8410501, Israel; 8 Department of Cardiothoracic Surgery, Lady Davis Carmel Medical Center, Haifa 3436212, Israel

**Keywords:** lung cancer, ground-glass opacity (GGO), artificial intelligence (AI) deep learning, computed tomography (CT), texture features: pixels

## Abstract

**Background/Objectives**: Ground-glass opacity (GGO) refers to areas of increased lung opacity on computed tomography (CT) scans. Distinguishing malignant from benign lesions using CT scans remains significantly challenging. This study aims to compare the performances of a linear multivariate statistical regression and an AI deep learning method in their abilities to predict GGO malignancy, given a set of pixel features extracted from CT scans. **Methods**: This retrospective study investigated patients from the Carmel Medical Center with findings of GGO nodules in their lung CT scans. Forty-seven consecutive patients were found to have either pure or part-solid GGO lesions, as defined by two independent radiologists. After manually segmenting the GGOs in the CT scans, pixel features were extracted using the MaZda software package, which analyzes six different image texture features. These textural variables were then compiled as input for the multivariate statistical regression. Additionally, an AI deep learning method, developed by our group and hosted on the cloud, was applied to the CT images containing the GGOs. **Results**: Among the 47 patients, 32 were diagnosed by pathology with malignant lesions and 15 with benign findings. Using the multivariate statistical regression, we identified 19 variables with statistically significant or near-significant differences through univariate analysis. In subsequent multivariate analyses, two independent variables that could distinguish between benign and malignant GGO lesions were identified: S(4,4)AngScMom (*p* = 0.012) and WavEnLH_s-2 (*p* = 0.008). The regression formula based on these two variables yielded a sensitivity of 91% and a specificity of 67% AUC: 0.8 (95% CI: [0.65, 0.94]). The AI deep learning model demonstrated a sensitivity of 100% and a specificity of 80% AUC: 0.96 (95% CI: [0.86, 1.00]). **Conclusions**: This proof-of-concept study demonstrates the superior performance of the AI deep learning model compared to the multivariate statistical regression, particularly in terms of sensitivity and specificity. However, given the small sample size, these results could potentially change with larger patient cohorts.

## 1. Introduction

Lung cancer is one of the leading causes of cancer-related mortality worldwide [1]. Despite advancements in diagnostic techniques and treatment methods, the survival rate for lung cancer patients remains relatively low [2,3,4]. One of the most relevant causes for this poor prognosis is the fact that the disease is often diagnosed at an advanced stage, which limits treatment options and reduces the likelihood of successful intervention [1]. Early and accurate diagnosis of pulmonary lesions, whether malignant or benign, is of high importance to tailor appropriate treatment for the patients and to improve their prognosis.

Lung cancer refers to a condition where there is abnormal tissue growth in the lung, in which abnormal cells multiply and grow uncontrollably, forming a tumor that interferes with normal lung function. The tumor can grow in the bronchi, the pleura, or in peripheral or central areas of the lung parenchyma [1]. The cancerous cells may spread through the lymphatic pathways and form metastases, reaching other organs in the body such as the liver, brain, bones, adrenal glands, and the contralateral lung, eventually leading to death [1]. Lung cancer is the most common malignancy worldwide for both genders, with nearly two and a half million new cases per year as of 2022, according to the International Agency for Research on Cancer (IARC), out of twenty-two million new cancer cases during that year [5]. It is estimated that one in five men or women may develop cancer during their lifetime. In addition, one in nine men and one in twelve women could die as a result of this disease. Lung cancer is a deadly malignancy responsible for 18.7% of cancer deaths worldwide [1,5]. The main risk factors for lung cancer are smoking, exposure to passive smoking, air pollution, ionizing radiation, genetic predisposition, and a history of previous chronic lung disease such as Chronic Obstructive Pulmonary Disease (COPD) [1,6]. Lung malignancies are divided into two main types according to the histology of the tumor cells: small cell lung cancer (SCLC) and non-small cell lung cancer (NSCLC). SCLC is an aggressive tumor, with an incidence rate of about 15% among all lung cancer cases [7]. Tobacco smoking is considered to be the main cause of SCLC. NSCLC accounts for over 80% of all lung cancer cases and includes two main types of tumors. The most common type of NSCLC is adenocarcinoma, often presenting as peripheral lung nodules, and the second most common is squamous cell carcinoma [6,7]. Even though pulmonary nodules and ground-glass opacity lesions (GGOs) are frequently discovered on lung CT scans, clinically significant GGOs tend to be malignant in more than 50% of cases [8,9,10].

The stage of the disease, also known as the staging of the tumor, assesses its spread in the body and affects the patient’s survival; the more widespread the tumor, the higher its stage. The stage of the disease is determined by three main components: the T component, which refers to the size and location of the tumor in the lung; the N component, which represents the involvement of lymph nodes in the disease; and the M component, which symbolizes the presence of distant metastases of the tumor. Generally, when there is involvement of lymph nodes, the tumor is in a locally advanced stage (stage 3), and when the tumor develops metastases and involves other organs, it is in an advanced stage (stage 4) [7,8]. One of the main reasons for high mortality rates is the detection of lung cancer at advanced stages—over 75% of patients are diagnosed at stage three/four of the disease, due to the scarcity or absence of symptoms in the early stages. The indications of lung cancer may include persistent coughing without improvement over time, hemoptysis (bloody sputum), chest pain that worsens with deep breathing, and shortness of breath. Less commonly, hoarseness, unexplained weight loss, fatigue, and other various symptoms may also occur [1,9]. In addition, the intensity of symptoms varies from person to person and can range from having very severe symptoms to being totally asymptomatic, thereby complicating diagnosis [9]. Despite a significant improvement in survival in recent years, the five-year survival rate for patients with lung cancer of all types is still only around 19% [10]. Early detection of lung cancer increases the chances of survival, improves prognosis, and significantly contributes to tailoring more effective medical treatment [11].

Diagnosis of lung cancer is based on appropriate imaging and lung sampling, which together determine the presence of the disease and the clinical stage of the disease from which the patient’s treatment policy is derived. Computed tomography (CT) scans are currently the primary imaging modality used in the identification and evaluation of lung cancer [7]. This modality offers detailed imaging of lung structures, including the lung parenchyma, blood vessels, bronchial tree, mediastinum, and pleura. When lung cancer is suspected, it is sampled to diagnose whether it is benign or malignant and to classify its type [7]. Most often, the sampling is done using one of three approaches: bronchoscopy, under CT guidance, or during surgery [12,13]. Each method has advantages and disadvantages that are taken into account, such as the degree of risk of complications for the patient, the accessibility of the finding given its location in the lung, and the amount of obtainable material in the sample, in comparison to the complete resection of the entire lesion. In addition to tissue diagnosis, the clinical stage of the disease must be determined in order to decide on treatment policy. Clinical staging is performed with a CT scan and a PET scan [14,15].

Understanding that earlier diagnosis increases the survival rate of patients, screening tests for the population have been offered in several countries [1,16] in recent years. In Israel, the Ministry of Health initiated a program for the early detection of lung cancer through CT screening [17]. This program is based on low-dose CT (LDCT) scans in individuals with an increased risk of developing lung cancer [7]. An LDCT scan is a special type of CT scan that uses a lower amount of radiation than a regular CT scan and allows for the identification of lesions and abnormalities in the lung parenchyma with acceptable accuracy using less radiation. Numerous randomized trials of LDCT screening have shown it to be much more sensitive than chest radiography [9]. Candidates for the screening tests are individuals with a history of smoking over 15 pack-years and over 55 years of age. The lung cancer detection rate is about 1% [16].

Once a lung lesion is characterized as malignant, and the disease is at an early stage, a surgery is performed during which the lesion is completely removed, and the lymph nodes draining the area in the lung where the tumor was located are sampled [18]. The lesion is evaluated by pathological and histological diagnosis to characterize all components of the lesion and thereby determine the accurate stage of the disease (pathological stage) [19,20].

One of the common incidental findings on CT scans is ground-glass opacity (GGO) lung nodules, which pose a significant diagnostic challenge [21]. A GGO lung nodule is a descriptive term for a non-specific CT scan radiological finding, showing increased lung tissue density while preserving its underlying bronchial structures and vascular blood vessels without obscuring these markings [11]. This finding can appear in a wide spectrum of pathological conditions, such as pulmonary infection, interstitial lung disease, pulmonary edema, and alveolar hemorrhage, but also as the initiation of lung adenocarcinomas [21], atypical adenomatous hyperplasia, adenocarcinoma in situ, minimally invasive adenocarcinoma, or invasive adenocarcinoma [12,13]. Although surgical resection of GGOs is often required for a definitive diagnosis, many GGOs are found to be benign or pre-malignant after surgery [14]. GGOs can be further classified as either pure GGOs or part-solid GGOs [21,22]. A pure GGO nodule does not have any solid component, and it is undetectable upon applying a soft tissue (mediastinal) window in a CT scan. A part-solid GGO lesion has a solid component that is visible on a soft tissue window in a CT scan. The ratio between the size of the solid component to the size of the complete lesion is referred to as the consolidation to tumor ratio (CTR). The larger the solid component and the higher the CTR, the greater the risk of malignancy [22]. Since lung cancer is a heterogeneous disease, it is hypothesized that tumor aggressiveness might be reflected in the micro-structural heterogeneity of the lesion, which could be captured by CT imaging [15].

Many models have tried to predict the risk of malignancy in these lesions and have included many variables such as nodule size, growth dynamics, percentage of the solid component, and the overall appearance of the opacity [23]. However, there is still no agreed-upon model that can predict malignancy with a high degree of accuracy, and save further investigations by interventional procedures (i.e., biopsy), or a prolonged follow-up of at least five years [24]. The yield of needle biopsy might be relatively low, which can lead to there being a high rate of false-negative biopsy results [25]. A recent observation of Faber et al. indicated that pathological staging has upstaged clinical staging of ground-glass nodules (GGNs) [26]. As a result, additional computer-aided diagnoses of chest CT scans are called for. Texture analysis is a branch of radiomics that quantifies image features such as intensity, distribution, and pattern of pixel values, potentially capturing information not visible on standard radiology interpretation [16]. Studies have shown that texture features can correlate with tumor invasiveness and patient prognosis [17,18,19]. The increasing use of AI and deep learning in medical imaging research has also led to many attempts to classify pulmonary lesions using complex algorithms [20,21,22,23]. These methods do not require manual feature extraction and can potentially learn relevant imaging biomarkers directly from the data. The use of artificial intelligence (AI) deep learning methods is needed to improve the accuracy of image analysis. Therefore, it is important to assess GGOs because these lesions may represent a very early malignant process. The analysis of visual images of GGO lesions can serve as an effective way to investigate and diagnose the lesion characteristics. This study aims to investigate, through the processing and technical analysis of GGO findings in CT scans of individuals with suspected lung cancer, whether it is possible to predict if the lesion is malignant or benign. The aim of the present study was to explore two different quantitative imaging approaches in classifying pure and part-solid GGOs as benign or malignant. Another aim of this study is to improve the diagnostic ability of GGOs and to suggest clinical care without the need for surgery, biopsy, and/or prolonged follow-up. Specifically, we applied a traditional radiomics approach (extracting texture features using the MaZda [27] software and performing multivariate statistical regression) and a deep learning approach (using a cloud-based convolutional neural network) to a cohort of surgically resected GGO lesions. We evaluated and compared their performances in a retrospective proof-of-concept study. By integrating established statistical techniques with modern AI, we hoped to gain complementary insights into the imaging characteristics that may differentiate malignant from benign GGOs. Additionally, the objective of the present study is to develop a prediction model for lung malignancy in a GGO lesion by trying to find technical characteristics and subtle patterns that are not discernible to the human eye, and which could distinguish between malignant tumors and non-malignant findings.

## 2. Materials and Methods

### 2.1. Study Design

This retrospective study included 47 consecutive patients of the Carmel Medical Center (CMC) who had pure or part-solid GGO lung lesions, based on preoperative CT scans, who were operated upon between 1 September 2015 and 31 December 2022. Strict inclusion criteria were applied: patients were ≥18 years old, had a pure or part-solid GGO nodule on CT, and underwent complete surgical resection of the lesion. The lesions were classified as malignant or benign after pathological processing and analysis. All eligible cases during the defined time frame were included, and no further selection bias was introduced; hence, the cohort represents the complete set of consecutive patients meeting the study’s criteria.

### 2.2. Ethical Considerations

The data relevant to this study were collected from the CMC medical records of the participating patients. All samples used in this study originated from the archives of the CMC Department of Pathology. Sample identification within the study was anonymous and based solely on a code number that cannot be directly linked to a specific patient. This study was in compliance with the principles of the Declaration of Helsinki and was approved by the CMC ethics committee (approval number CMC-0049-24 on 9 September 2024). Patient consent was waived due to the retrospective nature of the study.

### 2.3. Study Population

The inclusion criteria were patients over 18 years old who underwent surgery in the Thoracic Surgery Department at CMC for complete resection of a GGO lung lesion and had a CT scan performed prior to surgery. Exclusion criteria referred to patients with a GGO lung lesion who did not undergo complete resection of the finding or who did not have a CT scan performed prior to surgery.

### 2.4. Work Procedure

The current study utilized the fact that a computer reads a radiographic image as a matrix of pixels with different grayscale values. In this matrix, the numbers are integers that range from 0 (black) to 255 (white), allowing for the analysis of the texture of the pixels that make up the tumor and the stroma accompanying the tumor.

All CT images were high-resolution chest CT scans acquired on multi-detector CT scanners with thin slices (1.0–1.25 mm slice thickness). The in-plane image matrix was 512 × 512 pixels (typical for diagnostic chest CT), and images were analyzed in standard lung window settings (window ~1500 HU, level ~−600 HU).

In the current study, all CT images were acquired under similar conditions (axial orientation, high-resolution thin slices ~1 mm) across patients; hence, they were inherently consistent in geometry and scale. We did not explicitly resample or resize the images further; each region of interest (ROI) was analyzed at the CT’s native pixel resolution (which was comparable between scans). The spatial resolution was uniform (all images were high-resolution CT with sub-millimeter voxels), and no additional geometric transformations (rotations or magnifications) were required across the dataset.

Brightness normalization was carried out via MaZda texture software’s built-in preprocessing: images are converted to grayscale, and a normalization of gray-level intensity is performed before computing features [27]. All lesion images had their intensity histograms normalized (which mitigates scanner-to-scanner brightness differences). All images were analyzed using the same window level/width for consistency (lung window, approx. −600 HU center, 1500 HU width, as per standard chest CT).

No additional downsampling or upsampling was reported, so each image was processed at its native resolution (the pixel spacing across scans was on the order of sub-millimeter and did not require normalization). All scans had the patient in a supine position with similar axial orientation; therefore, no rotation or geometric alignment adjustments were necessary across the dataset.

For each patient, one representative CT slice containing the GGO nodule was used for analysis. The GGO lesion was manually segmented (outlined) on that CT slice by the investigators (with input from the radiologists). Two experienced radiologists independently identified and segmented each GGO (using dedicated software), and discrepancies were resolved by consensus to verify accuracy. The two board-certified radiologists initially identified and defined the GGO lesions (pure vs. part-solid) to ensure the correct region was targeted for segmentation. The manual segmentations encompassed the entire nodule, and a single operator performed the ROI delineation. The involvement of two radiologists in lesion identification provided a form of verification for the segment location.

For preprocessing, the CT images were input into the MaZda texture analysis software, which automatically performs gray-level normalization prior to feature extraction [27]. This means the lesion pixel intensities were normalized to a consistent scale (reducing brightness variations between scans) before computing texture features. No additional filtering or noise reduction beyond what MaZda’s standard pipeline does was applied. Because feature computation was confined to the segmented ROI (the GGO lesion), regions outside the lesion (background lung, annotations, etc.) did not contribute to the analysis. Thus, any extraneous image information (e.g., scanner text labels in margins) was effectively excluded by focusing on the ROI.

For geometric matching and irrelevant information removal, the current study’s approach focused on the manually segmented ROI. Only the pixels inside the outlined GGO lesion were used for texture feature extraction. Thus, no additional cropping was needed beyond selecting the lesion region. All ROIs were drawn in the axial plane (no rotation needed, since each CT is already in standard orientation). Furthermore, because each ROI was defined as the full tumor area, the “diagnostically significant region” was consistently included, and we did not have to pad or scale ROIs—each was analyzed at true size in the context of its source image. In summary, each image was preprocessed to a uniform standard: same resolution and orientation (native CT resolution), same intensity normalization and windowing, and analysis confined to the segmented lesion area (excluding any extraneous image content).

**Figure 1:**
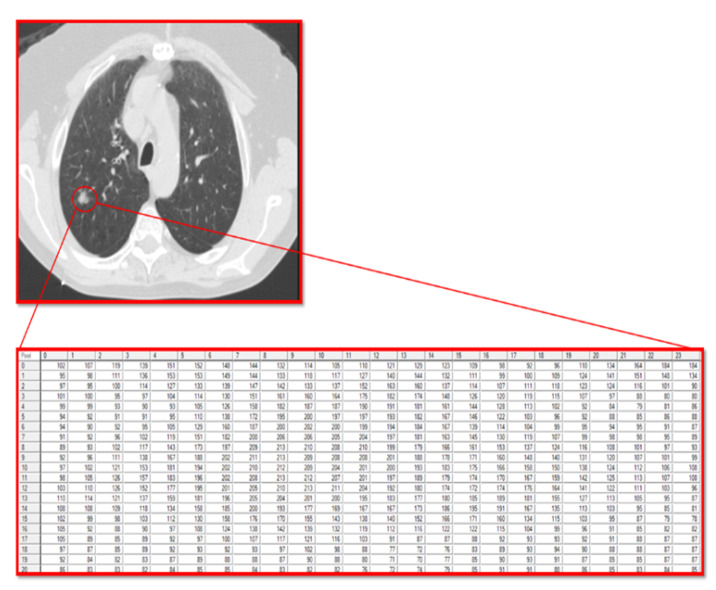
Lung CT scan with ground-glass opacity (GGO). The GGO lesion is marked with a circle, and the pixel matrix shows the tumor texture. A zoomed-in view of the grayscale pixel intensities used for texture analysis. In this matrix, grayscale values are integers that range from 0 (black) to 255 (white), allowing for the analysis of the texture of the pixels that make up the tumor and the stroma accompanying the tumor.

For texture analysis, a digital technology called MaZda (version 4.6) was used [27]. To calculate the texture variables of the GGO lesions in the radiographic image, an annotation of the tumor region was performed. Subsequently, the software calculated the texture variables (Figure 1).

**Figure 2:**
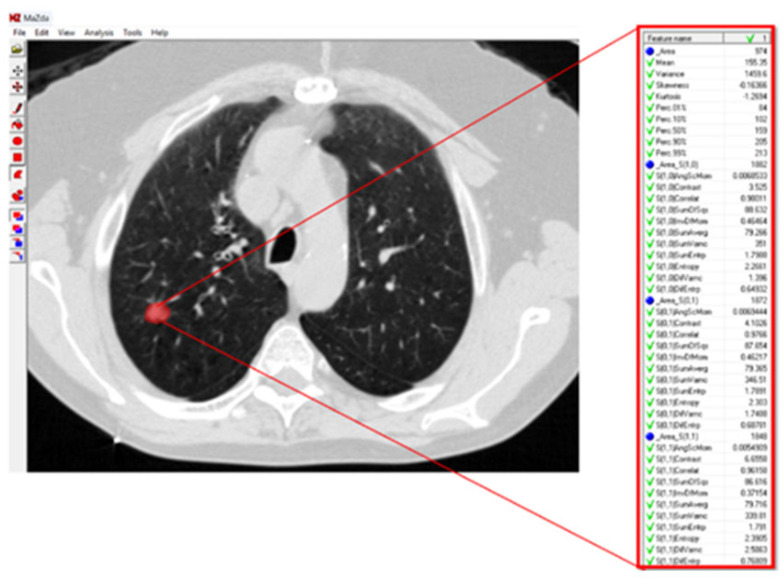
Lung CT scan with GGO texture analysis. About one-third of the variables extracted by the MaZda 4.0 software for the texture analysis of the GGO in this lung CT scan are presented. The red circle denotes the GGO region of interest.

The texture variables include pixel relationships, light intensity ratios, parameters of homogeneity in the image, and texture. All of these characterize the texture of image patterns and can be divided into groups according to how they are calculated and according to the type of information they provide about the texture being examined:

1. Texture variables calculated from the histogram of the distribution of light intensity values (grayscale) of the pixels that make up the image, or the pattern marked as an ROI within the image.
2. Texture variables that reflect the flow of light intensities of pixels along a specific vector (i.e., from one side to the other of the image, or radially between the center and the edge of the image) of the pattern in the image.
3. Texture variables obtained from calculations performed on a matrix called the Gray Level Run-Length Matrix, which is the result of a matrix transformation of the original matrix of pixels encoded by their light intensities. The Run-Length Matrix is based on calculating the frequency of neighboring pixels with the same light intensity.
4. Texture variables obtained from calculations performed on a matrix called the Gray Level Co-Occurrence Matrix, which is the result of another matrix transformation of the original matrix of pixels encoded by their light intensities. The Co-Occurrence Matrix is based on calculating the frequency of combinations of pixels with different light intensities.
5. Texture variables obtained from building an autoregression model that tries to predict the value of a specific pixel based on the values of neighboring pixels.
6. Third texture variables obtained from post-matrix transformation calculations (involving wavelets) of yet another matrix transformation of the original matrix of pixels encoded by their light intensities (Figure 2).
7. These texture features quantify different aspects of heterogeneity in the GGO region, such as intensity distribution, spatial frequency content, and structural patterns.

A matrix is a mathematical object containing a 2D array of numerical values that can be manipulated by various algebraic operations. A matrix transformation is a special type of matrix-to-matrix function; that is, it takes in a matrix as its input and returns a matrix as its output. Specifically, the output matrix produced from a matrix transformation of a given input matrix is defined to be equal to the matrix product of a fixed-value transforming matrix and the input matrix. In addition, since we want the input and output matrices to have the exact same dimensions, we can necessarily state that the transforming matrix should be a square matrix.

### 2.5. Statistical Analysis

The Kolmogorov–Smirnov test (hypothesis testing for the type of sampling distribution) was applied to process continuous variables to examine the normality of the variables (null hypothesis: sampling distribution is normal; alternate hypothesis: sampling distribution is not normal) within the different groups. Univariate comparison between parametric groups was performed using the unpaired *t*-test. Subsequently, a statistical classifier based on linear logistic regression for the selection of independent variables was used. Using the regression coefficients obtained from the statistical analysis of the texture of the GGO lesions, a formula was developed to predict GGO malignancy. A receiver operating characteristic (ROC) analysis was performed in order to determine the optimal cutoff point for the Discriminant Score (DS). The area under the curve (AUC) was calculated in order to assess model performance. The statistical analysis was performed using SPSS version 26 software (IBM, Armonk, NY, USA).

In addition to the statistical analysis, an analysis of all of the aforementioned CT images was performed using an AI deep learning model, with the use of a free Google application, known as Google Teachable Machine (Vestion 2.0) [28]. The Google Teachable Machine model is a deep convolutional neural network (CNN) classifier based on transfer learning. Specifically, it uses Google’s pre-trained MobileNet architecture as the feature-extraction backbone. MobileNet is a lightweight CNN designed for efficient image recognition, consisting of dozens of layers of convolution and depth-wise separable convolution (organized into ~17 bottleneck blocks) followed by a fully connected output layer. In our application, the MobileNet’s final layer was replaced with a new dense layer for binary classification (malignant vs. benign). Thus, the Google Teachable Machine model network includes the many convolutional layers of MobileNet (on the order of ~50 layers in total, counting all conv/activation layers) plus one output layer (a sigmoid neuron for the two-class output). The Teachable Machine platform handles this architecture internally. It is a MobileNet-based CNN with a custom classification head.

Training was carried out for 50 epochs (the default setting on Teachable Machine). During training, the platform used a batch size of 16 and a learning rate of 0.001 (defaults that we did not override). The loss function employed was the standard cross-entropy loss for classification. In this two-class case, the model was optimized with binary cross-entropy loss, which is the typical choice to measure classification error. (Cross-entropy loss penalizes the model when its predicted probability diverges from the true class label, and is the appropriate objective for training a classifier.)

Google Teachable Machine’s standard image model (a headless MobileNet CNN) was employed and trained for 50 epochs using the Adam optimizer and categorical cross-entropy loss.

**Figure 3:**
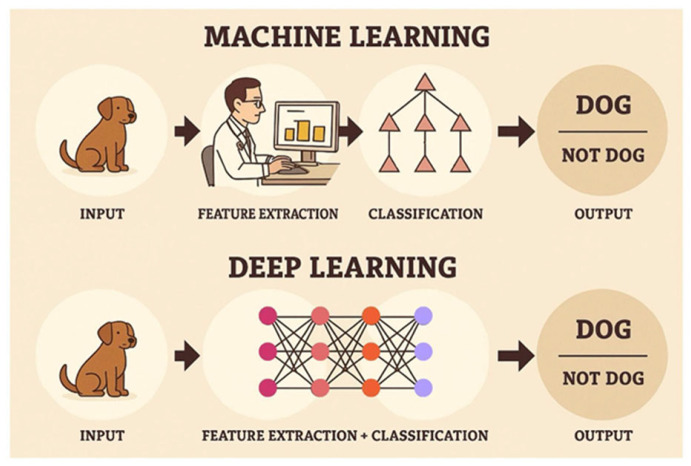
Machine learning model and deep learning model. This figure demonstrates the differences between the machine learning model and the deep learning model used by the Google AI model developed for this study. The machine learning model involves two separate steps: first, feature extraction, then classification, followed by an output. The deep learning model has one step of combined feature extraction and classification done to produce an output.

Unlike the traditional statistical analysis method, in which numerical features must first be measured and collected using measurement algorithms that are then fed into a network of “neurons” (formulas), the AI deep learning model is based on an initial analysis that uses convolutional filters that automatically extract features from the images, that are then subsequently fed into the model’s “neural network”. This network iteratively corrects the parameters of the “neurons” (i.e., formulas) in order to achieve the maximum likelihood that allows for the best reasonable prediction of the diagnosis (Figure 3).

The majority of the CT scan images were used for the AI deep learning model’s training, whereas a separate subset of the CT scan images was reserved for cross-validation. For training and validation, the CT images were randomly split into a training set (70% of images) and a validation set (30% of images). Cross-validation was performed, repeating this 70/30 split and processing multiple times in order to ensure model robustness. The model’s performance was assessed by evaluating the sensitivity and specificity on the validation set. No image augmentation (e.g., rotation, scaling, or flipping) was performed. In the current study, we did not apply any explicit balancing techniques (such as oversampling the minority class or using class-weighted loss) to address the class imbalance. The training and validation sets preserved the natural distribution of 32 malignant and 15 benign cases. During the random 70/30 splits, we ensured each split contained a representative mix of classes, but we did not implement additional rebalancing; hence, the class imbalance was unmodified.

## 3. Results

### 3.1. Demographic and Clinical Characterization

Data from 47 patients were collected in this study, and their characteristics are presented in Table 1. The patients included 17 men and 30 women. The median age of the patients was 70 years (range: 43-82 years). 23% of the patients were active smokers. All patients underwent surgery for lesion removal, followed by pathological characterization by histopathological examination to classify the lesion as malignant or benign. 68% (32/47) presented with cancerous tissue, of which 53% (25/47) were diagnosed with adenocarcinoma and 15% (7/47) were classified as adenocarcinoma in situ; 32% (15/47) had benign lesions. 47% (22/47) of the patients were diagnosed with pathological stage IA1, and another 47% (22/47) were diagnosed with stage 0. None of the patients had lymphovascular invasion. From the pathological reports, the median size of the invasive part was found to be 5 mm (range 0–20 mm), and the median overall size of the malignant lesions was found to be 10 mm (range 5–25 mm).

### 3.2. Texture Analysis of GGO Lesions

Using the MaZda software for texture analysis of GGO lesions, a large number of variables were generated. 19 of the variables showed statistical significance/near-significance (*p*-value < 0.1) in the univariate analysis between malignant and benign GGO lesions. Because the current study is a small proof-of-concept study, and many variables were screened in univariate analysis, a common practice of noting trends when 0.05 < *p* < 0.1 was followed. *p* < 0.1 was considered a statistical trend (‘near-significant’) given the exploratory nature of this analysis. Features with *p* < 0.1 in univariate testing were carried forward as suggestive trends, with the rationale of limited sample size and exploratory aim. These features included parameters from the Gray Level Co-Occurrence Matrix, the autoregressive model, and wavelet-transformed images. Table 2 presents the different variables with statistical significance levels of each variable for the prediction of malignancy.

After performing the multivariate analysis by logistic regression, two variables emerged as independent and unrelated. The most significant features in the univariate analysis were S(4,4)AngScMom and WavEnLH_s-2. Table 3 shows these two variables and their levels of significance. In the multivariate logistic regression model, only S(4,4)AngScMom (*p* = 0.012) and WavEnLH_s-2 (*p* = 0.008) remained as independent predictors distinguishing malignant from benign GGOs.

### 3.3. Variables and Their Significance

#### 3.3.1. S(4,4)AngScMom—Second Angular Momentum

The term “angular” refers to the fact that texture intensity values are distributed at different angles. S(4,4) indicates that this feature is calculated using a specific window size or distance (4 pixels), and it is based on the calculation of the second moment. In texture analysis, the second moment refers to the variance of intensity values. These data are related to homogeneity, where a low value indicates high contrast and a high value indicates low contrast.

When the value is high, it indicates that the texture of the lesion is relatively uniform in intensity values. This indicates a smooth or homogeneous texture in the lesion, meaning that the tissue structure is fairly consistent, with little contrast or sharp edges. Higher values are associated with benign lesions. When the value decreases, the texture is more heterogeneous, with noticeable variance in intensity values; that is, there are areas of high contrast or irregularities in the tissue structure. Lower values suggest malignant characteristics.

#### 3.3.2. WavEnLH_s-2—Wavelet Energy Low-High Frequencies

This variable involves an analysis technique that decomposes the image into different frequency sub-bands to capture details at various levels of resolution.

This variable refers to the wavelet energy in the low-high frequency band at a specific scale (the second scale). This sub-band captures information in both low frequencies (overall shapes) and high frequencies (details), providing insights into the texture of the lesion at multiple levels of resolution.

When the value is high, it indicates that the lesion contains many high-frequency details, along with smoother areas. This suggests irregularities such as boundaries, calcifications, or more complex textures in the lesion. Higher values are associated with benign lesions.

When the value is low, it indicates that the lesion contains few high-frequency details (i.e., it may be a smoother texture with less sharp edges or little contrast). The lesion will often be homogeneous or smooth. Lower values are associated with malignant lesions.

These features help to distinguish between different types of lesions, as texture patterns can indicate tissue characteristics and may be suggestive of the nature of the lesion (i.e., whether it is malignant or benign). For example, malignant lesions often show more heterogeneous texture patterns with lower WavEnLH_s-2 values, while benign lesions tend to have more homogeneous patterns with higher S(4,4)AngScMom values.

### 3.4. The Discriminant Score (DS) Formula Calculated from the Regression Coefficients

The resulting discriminant formula based on these two variables (S(4,4)AngScMom; WavEnLH_s-2) was used to compute a malignancy score for each case. This formula used the slope and constant values from Table 3.

DS = 4.693 − (S(4,4)AngScMom × 149.096) − (WavEnLH_s-2 × 0.04)(1)

DS = β_0_ − β_1a_(S(4,4)AngScMom) − β_1b_(WavEnLH_s-2) (2)

β_0_ denotes the intercept; β_1a_ denotes the slope of S(4,4)AngScMom; β_1b_ denotes the slope of WavEnLH_s-2.

**Figure 4:**
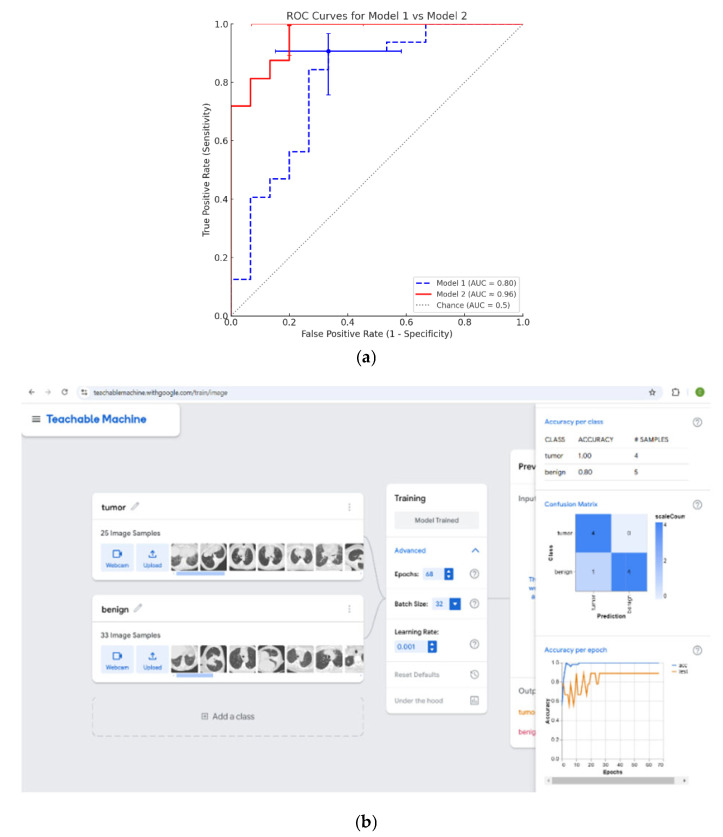
ROC curves and image analysis. (**a**) Comparative ROC curves for Model 1 (MaZDA-based ML) vs. Model 2 (Google Teachable Machine CNN): Model 2’s curve (red solid) dominates Model 1’s (blue dashed), corresponding to a higher AUC. The dot and square show the sensitivity/specificity operating points for Model 1 and Model 2, respectively, with 95% CI error bars. The diagonal dotted line is the no-discrimination reference (AUC = 0.5). Key Performance Metrics: Model 1, sensitivity: 91%, specificity: 67%, AUC: 0.80 (95% CI: [0.65, 0.94]). Model 2, sensitivity: 100%, specificity: 80%, AUC: 0.96 (95% CI: [0.86, 1.00]). (**b**) Performing the analysis using AI Deep Learning: This image was taken from the output display of the CT image analysis (including the lesions). The image shows only a sample portion of the images.

An analysis of a ROC curve can be used in order to determine the optimal cutoff point for the DS, which was found by the logistic regression. ROC analysis of this score demonstrated an optimal threshold for identifying malignant GGOs. The cutoff point yielded a sensitivity of 91% (95% CI: 85–97%) (where the DS value is higher than this cutoff point, there is a 91% certainty that the lesion is malignant) and a specificity of 67% (95% CI: 50–84%) (where the DS value is lower than this cutoff point, there is a 67% certainty that the lesion is benign) (Figure 4a). The AUC for this model was 0.8, indicating fair discrimination.

For the MaZda machine learning model, given the sample sizes (32 malignant, 15 benign cases), the 95% CI for this model’s sensitivity is 76–97%, and for specificity is 42–85%.

Since it is practically impossible to obtain sensitivity and specificity values that are both 100% simultaneously, it is preferable to have a high sensitivity rather than to miss actual positive cases that could mistakenly be classified as benign by the computed model system; that is, in this case, it is acceptable that false positive cases undergo diagnostic biopsy. However, this statistical analysis provided a sensitivity that is still not optimal. Therefore, the lesions were also examined with the help of an AI-based system in an AI deep learning model developed through Google applications and offered free to online users.

### 3.5. Performing the Analysis Using AI Deep Learning

This AI deep learning model was first trained with an analysis of most (70%) of the pre-classified images in order to build a “knowledge” algorithm that can later be tested for cross-validation purposes, reserving a subset (30%) of images from each category for testing purposes. The reserved images were then used for cross-validation in order to test the AI deep learning model that was trained on the majority of the images. The deep learning model trained on the original set of 47 images (with repeated cross-validation) achieved a sensitivity of 100% and a specificity of 80% on the validation sets. The 95% CIs are broader in this small sample: sensitivity 71–100%, specificity 38–96%. Despite the wide intervals (due to the limited N), they still indicate that the deep learning model’s sensitivity is statistically high.

Comparative ROC Curve Analysis: Figure 4a overlays the ROC curves of the MaZda-based ML model (Model 1) and Google Teachable Machine CNN model (Model 2) on the same axes. Model 2 (red solid line) shows a ROC curve closer to the top-left corner, indicating superior discriminative ability (AUC: 0.96, 95% CI: 0.86–1.00) compared to Model 1 (blue dashed line, AUC: 0.80, 95% CI: 0.65–0.94). The gray diagonal line represents chance performance (AUC = 0.5). Model 2 achieved 100% sensitivity and 80% specificity (operating point at true-positive rate (TPR) 1.0, false-positive rate (FPR) 0.20), whereas the MaZda-based model (Model 1) reached 91% sensitivity and 67% specificity (TPR 0.91, FPR 0.33). These operating points are marked on the curves (square for Model 2, circle for Model 1), with error bars showing the 95% confidence intervals for sensitivity (vertical) and specificity (horizontal) at those points. The comparatively narrow error bars for Model 2 reflect more certainty in its performance, while Model 1’s wider error bars (especially for specificity) indicate greater uncertainty due to the smaller sample of negative cases.

The higher AUC and sensitivity of Model 2 indicate that the CNN approach provides better overall diagnostic accuracy, correctly identifying all malignant cases in the study (no false negatives) while also reducing false positives relative to Model 1. This improvement is visualized by Model 2’s ROC curve bowing further upward and leftward, yielding a larger area under the curve and a point at (FPR = 20%, TPR = 100%) that lies above Model 1’s operating point.

On the right side of Figure 4b, there are two graphs; the upper graph displays the accuracy of the classification of the images that were used for the iterative learning of the model’s “neurons” (formulas). Notably, after a number of iterations (epochs), the model fully classified, with 100% accuracy, all of the images according to the diagnostic categories. The lower graph displays the accuracy of the classification of the images that were used to test the model, i.e., for cross-validation.

It can be seen from the graph (right side of Figure 4b), as well as from the binary table above it, that the classification level of the testing images was very high, with a sensitivity of 100% and a specificity of 80%. The model correctly identified all malignant cases and had a false-positive rate corresponding to 20% of benign cases. The overall accuracy of the deep learning model was 89%. The AUC of the deep learning model’s ROC curve was 0.96, exceeding that of the logistic regression model. These results suggest that the AI model outperformed the traditional statistical model in this dataset. This model can allow for the prediction of all malignant cases; however, there will also be a number of benign cases that will likely be mistakenly identified as malignant. This is not a problem because it is preferable to perform a biopsy (which is not life-threatening) for histological diagnosis, rather than miss a positive case by a false negative. The AI deep learning model that we developed through the use of Google applications is stored in the cloud. The AI deep learning model has a link that can be sent via email to other researchers upon request, who can then use it to examine CT images of patients in other medical centers.

## 4. Discussion

This study aimed to find a method of prediction that is capable of applying a mathematical formula, in order to characterize pulmonary GGO lesions as either malignant or benign, thereby avoiding surgeries and biopsies for benign cases. Two quantitative approaches were applied: a radiomic/statistical model and a deep learning model. Both methods utilized CT imaging data but differed fundamentally in feature extraction and modeling. During the research, the processing and analysis of pixel features of GGO lesions from CT scans of patients with suspected lung cancer was performed with the use of the MaZda software, which interpreted the lesion on the scan as a matrix of numerical values in different texture variables. The analysis of the texture features using MaZda treated each GGO on CT as a matrix of pixel intensities. This approach allowed for the determination of image heterogeneity through multiple texture features. Subsequently, a univariate analysis was performed in order to find texture variables with statistical significance (*p*-value < 0.05) or near significance (0.05 < *p*-value < 0.1) that are able to distinguish between CT scans with GGO lesions characterized as either malignant or benign. The univariate analysis identified 19 texture features with potential significance. The 19 statistically significant/near-significant texture variables were processed through a multivariate analysis in a statistical linear regression model, out of which two independent variables were found to predict the diagnostic groups. The multivariate regression revealed that two variables—S(4,4)AngScMom (an angular second moment) and WavEnLH_s-2 (a wavelet energy feature)—could independently differentiate between malignant and benign GGOs. Through this analysis, a formula was developed that takes on these two variables as algebraic arguments, in order to predict the characterization of a lesion as either malignant or benign. Incorporating these into a logistic regression formula yielded a high sensitivity (91%) but moderate specificity (67%). After that, an analysis of the ROC curve was performed in order to determine the threshold that can distinguish between a malignant and a benign lesion [29]. This threshold was determined according to different sensitivity and specificity measures. Sensitivity and specificity are measures of a diagnostic test’s performance. Sensitivity is defined to be the probability of identifying an individual who does have a true malignant lesion condition as actually having that malignant condition, according to a probabilistic test. Specificity is defined to be the probability of identifying an individual who does not have a condition, e.g., a true benign lesion, as actually not having that condition, e.g., benign, according to a probabilistic test [30] (Table 4).

Lesions that receive a post-processing numerical value above the threshold will be classified as malignant, while lesions that receive a post-processing numerical value below the threshold will be classified as benign. In this situation, getting a high sensitivity value is more important than getting a high specificity value. It is strongly preferable not to miss malignant cases by mistakenly classifying them as benign in the system. A higher sensitivity allows for the classification of as many lesions as possible as malignant out of those that are truly malignant. In contrast, a higher specificity allows for the classification of as many lesions as possible as benign out of those that are truly benign. If the specificity is lower, then there will be relatively more false positive cases (i.e., where there will be lesions that are benign yet are initially classified as malignant, and will thus undergo diagnostic biopsy, and only then be properly classified as benign). Hence, there is a clear need to find a threshold where the sensitivity is as high as possible, even if the specificity is lower. In the context of GGOs, high sensitivity is particularly desirable to ensure malignant cases are not overlooked, even if it means accepting a lower specificity.

In the traditional statistical analysis method, the resultant cutoff point was a sensitivity of 91% and a specificity of 67%. In comparison, the AI deep learning model produced a cutoff point with a sensitivity of 100% and a specificity of 80%. In other words, the AI deep learning model in the current research dataset narrowly overperformed the traditional statistical analysis method for all practical intents and purposes. However, it is important to note that conducting a proper statistical analysis can noticeably aid in the understanding of the statistical process, which, in this case, allows us to see texture changes via the values of specific variables that are able to differ between malignant and benign cases. Specifically, a statistical analysis enables a better understanding of which textural parameters are the deciding variables between the classification of lesions, and thus, in our case, contributes to a better understanding (at the biological level) of the morphology of the lesion. This allows for a future possibility for these texture differences to be studied by a clinician who seeks to examine CT images in a preliminary analysis (with the naked eye).

The findings of the current research are in line with prior studies showing that convolutional neural networks can effectively classify GGOs. Traditional computerized statistical analysis programs are able to distinguish between benign and malignant GGOs, as well as invasive or non-invasive cancer [31]. Heterogeneity observed in malignant tumors may indicate changes in the tissue structure, representing uneven cell density, degeneration, necrosis, hemorrhage, etc. [31].

CT images of GGNs were passed through an AI deep learning model in order to correctly predict between benign and malignant lesions [32]. An analysis of the ROC curve and its AUC assessed the predictive capabilities of the model developed by Yang et al. [32]. Yang et al. reported an AUC equal to 0.92 for their AI deep learning model, discriminating benign from malignant GGNs, which is comparable to the results of logistic regression of our AI deep learning model (AUC = 0.96) [32].

The classification of GGNs by Deng et al. was performed by employing a novel feature fusion algorithm. It included the evaluation of clinical and morphological features of GGNs on chest CT scans, as well as the extraction of whole-lung radiomics features, followed by deep convolutional neural networks. An attention mechanism was used to integrate the deep features. This AI deep learning model demonstrated a high performance, with an AUC value of 0.9 [33].

Bin et al. used an AI deep learning method, based on radiomics for pulmonary GGN classification, in order to predict invasion in early-stage adenocarcinoma. That study model, Random Forest (RF), employed texture features and achieved significant results in distinguishing invasive from non-invasive lesions [31].

Tasnim et al. used image processing methodologies on nodules in order to detect lung cancer, based on 3D CT scan outcomes and convolutional neural networks. Their work yielded an 80% accuracy [34].

A recent review by Duranti et al. reported the advantages of AI algorithms in predicting the risk for lung cancer via the usage of software developed for processing GGOs on CT images [35]. Liu et al. further assessed the potential of AI in diagnosing the risk of malignancy of GGOs through ^18^F-FDG PET CT studies by using convolutional neural networks. The segmentation prediction model achieved a sensitivity of 84.91%, an accuracy of 84.81%, and a specificity of 84.62% [36]. Lai et al. designed an algorithm for image processing using AI deep learning based on a 3D high-resolution representation of FDG PET-CT. This model automatically classified pulmonary nodules with the following prediction performances: an AUC of 0.781, a sensitivity of 89.9%, a specificity of 54.5%, and an accuracy of 79.4% [37].

An additional recent review evaluated algorithms of AI deep learning models for the detection of GGO nodules on chest CT scans. The highest accuracies for image classification achieved by AI models were reached by DenseNet AI (99.48%) and then WoANet AI (98.78%). However, this review did not focus on determining the GGOs’ potential for malignancy; rather, this review acknowledged the computer-aided detection of the GGOs [38].

The results of the current “proof-of-concept” study indicate that both traditional texture analysis and an AI deep learning model can effectively predict the malignancy of GGO lesions in CT imaging. The radiomic approach, by the use of the MaZda software, identified two independent texture variables, namely S(4,4)AngScMom and WavEnLH_s-2, that definitively distinguished between malignant and benign GGOs. These findings align with prior studies demonstrating the use of texture variables and wavelet-transformed image analysis in lesion characterization [39].

Another study supports the notion that AI and texture analysis can capture imaging biomarkers associated with tumor biology. The research by De Oliveira et al. [40] on oropharyngeal carcinoma investigated patterns of CT radiomic imaging-derived features and demonstrated that CT texture patterns could differentiate HPV-positive from HPV-negative oropharyngeal cancers. The results showed that the use of CT texture could indicate HPV+ status from HPV- lesions; however, it was not correlated with histological differentiation grade. The findings that CT texture patterns could reflect underlying tumor characteristics in oropharyngeal squamous cell carcinoma (specifically HPV status), reinforcing the potential of CT texture analysis as a non-invasive oncologic imaging biomarker [40]. That study supports the notion of applying texture-related data to detect benign vs. tumor tissue in CT scans analysis. CT texture/radiomic features serve as valuable imaging biomarkers for tumor characterization across different cancer types. This reinforces our study’s approach of using texture-derived parameters for predicting GGO malignancy and lends further rationale to using texture analysis in lung oncology.

The AI deep learning model developed for the current study achieved a sensitivity of 100% and a specificity of 80%, overperforming the traditional logistic regression method, with an AUC of 0.96. This supports recent research reporting on the use of AI deep learning algorithms in classifying GGO lesions. For instance, Yang et al. employed an AI deep learning model for predicting between benign and malignant GGOs, with an AUC value exceeding 0.90, thus emphasizing its clinical applicability [32]. Similarly, Deng et al. used AI deep learning in order to incorporate clinical and morphological features into a system based on a convolutional neural network, achieving a high accuracy through a novel feature fusion approach [33].

In the current study, the logistic regression model with radiomic inputs yielded a sensitivity of 91% and a specificity of 67%. While this is an underperformance of the convolutional neural network, this approach demonstrates features that can be interpreted in various ways and that offer clinical insights that are in agreement with an AI comprehensive review of lung cancer risk stratifications by Duranti et al. [35].

Moreover, the findings of the present study are in line with those of Bin et al., who demonstrated that an AI deep learning model can use radiomic features in order to predict invasive behavior in early-stage adenocarcinomas [31]. This further supports the methodology and findings of the current study, thus highlighting the potential role of texture analysis in non-invasive diagnostic systems. On the other hand, the specificity values of this study are lower than those that were achieved in PET-CT-based AI models, such as the AI models developed by Liu et al., who reported a specificity value of 84.6% by using FDG uptake patterns and convolutional neural networks [36]. Notably, while PET-CT adds valuable metabolic information, CT-based AI models (similar to those of the present study) have the advantages of being both more readily available and more cost-efficient, in addition to exposing the patient to less radiation. Shah et al. reported a very high accuracy with the DenseNet and WoANet AI models, using AI deep learning in order to detect GGOs, though their focus was on detection rather than on malignancy prediction [38]. This distinction emphasizes the uniqueness and relevance of this study.

It is important to note the complementary nature of the two methods. While the AI model achieved higher overall accuracy, the statistical regression model provides interpretable features that correlate with tumor heterogeneity. The two independent features identified (S(4,4)AngScMom and WavEnLH_s-2) could be further studied by clinicians to understand the biological underpinnings of GGO morphology. In fact, these texture variables describe aspects of homogeneity and frequency content in the lesion, which might correspond to histological complexity. The transparency of the regression model could thus yield insights that are not immediately apparent from the “black box” of a neural network.

Explainable artificial intelligence techniques such as Grad-CAM or LIME could be added to the deep learning model to help users comprehend how the AI model decides whether the lesion is classified as benign or malignant by giving the reasons why the deep learning model reached its decision. The explainable artificial intelligence techniques contribute to the trustworthiness of the machine learning [41]. Overall, the current study complements existing evidence supporting AI-assisted diagnostic tools and highlights the practical feasibility of implementing such tools in routine care. This proof-of-concept study indicates that both radiomic texture analysis and AI deep learning can effectively predict malignancy in GGO lesions on CT. These findings align with prior literature showing that image heterogeneity (as quantified by texture features) and AI algorithms are promising tools in lung cancer imaging [31,32,33,34,35,36]. Notably, although our AI model performed well, it is paramount to recognize the limitations of our study. Future studies with larger patient cohorts and external validation are required in order to refine these AI models and also improve generalizability.

### Limitations of this Study

In this study, which is designed under the “proof-of-concept” format, a total of 47 patients were included. In order to definitively prove the core theory of the current study, the actual number of patients included (47) is considered to be fairly low, yet still sufficient. The reason for not using data augmentation (e.g., rotation, scaling, or flipping) in the current study was to adhere to the original source files. This may limit the model’s robustness to variations in image acquisition and patient positioning. We acknowledge that augmentation can help in small datasets, and this is a potential area for future work. Future research could consider incorporating augmentation techniques with methods like oversampling or weighted-loss functions to mitigate any bias from class imbalance and to enlarge the effective dataset. In addition, the dataset was imbalanced (32 malignant vs. 15 benign), and we did not apply any class-rebalancing methods such as oversampling or weighted-loss functions. Class imbalance may bias the model toward the majority class. Future work could explore techniques like oversampling the minority class or using weighted-loss strategies to address this, potentially improving the model’s ability to recognize less common classes. However, the small sample size of this study limits the strength of the conclusions regarding the sensitivity and specificity of both the traditional method and the AI model as diagnostic tools. Further future studies with larger sample sizes are needed in order to fully cement the conclusions of the current study as being definitively true; larger patient cohorts and external validation are required to confirm the current study findings. Moreover, since the segmentation of GGO lesions in the various CT scans was done manually by radiologists, it is important to note that there may be inaccuracies in the lesion markings. We acknowledge that no second independent drawing of the ROI was done; the potential for minor human error in manual contouring is noted as a study limitation. Consequently, a feasibility proposal can be implemented for further research on a large number of patients in order to improve both the traditional statistical analysis method and the AI deep learning model developed for this study. This study was conducted at a single institution, which may limit external applicability. In future work, we intend to test our multivariate regression and AI models on an independent cohort (e.g., from another institution or public repository) to confirm their performance and generalizability. We plan to validate our findings on independent external cohorts to ensure the model’s robustness across different scanners and patient populations.

## 5. Conclusions

In this study, two distinct quantitative methods are presented for the purpose of predicting malignancy within GGO nodules appearing on CT scans. They show that texture-based multivariate analysis and a convolutional neural network can each differentiate malignant from benign GGOs. Specifically, in GGO nodules, there is generally no urgency to surgically operate because their rate of growth is relatively slow. Importantly, GGO lesions typically have a relatively indolent behavior; therefore, non-invasive tools that can predict malignancy could inform decisions about surveillance versus surgery. The presence of a model may contribute to improved accuracy in diagnosis as an additional objective “tool” in the experts’ arsenal of “tools” for evaluating and monitoring patients with GGO lesions. In practice, identifying benign GGOs can avoid unnecessary biopsies, while identifying malignant GGOs ensures timely intervention. Identification of GGO benignancy is crucial in order to avoid biopsies that could be fairly inaccurate in some patients, while identification of GGO malignancy is crucial in order to avoid missed diagnosis in other patients. In this study, two separate prediction methods are presented: The first method for predicting GGO malignancy with the texture analysis approach (using MaZda-extracted features in a logistic regression model) utilized a traditional multivariate statistical analysis and achieved a sensitivity of 91% and a specificity of 67%. The second method for predicting GGO malignancy utilized an AI deep learning model with a sensitivity of 100% and a specificity of 80%. It is important to note that, in the first method as mentioned above, the actual multivariate analysis involves the usage of a formula that takes in as its input texture variables that are identifiable as independent parameters (in this study: S(4,4)AngScMom and WavEnLH_s-2) and then returns as its output a probabilistic prediction of the nature of the GGO lesion as either malignant or benign that is parameterized by the stated statistical results of the first method, as mentioned above. Although the sensitivity and specificity values of the multivariate analysis method are both lower in comparison to the AI deep learning model, it is still advantageous to use the multivariate analysis method. In particular, more can be learned about the different processes for calculation within the multivariate analysis formula, which can then potentially be used within the clinical setting in the future. The deep learning model had higher overall accuracy; nonetheless, the radiomic approach provided interpretable features (S(4,4)AngScMom and WavEnLH_s-2) that may offer clinical insight. Moreover, if given a larger number of participating patients in the future, it is possible that the multivariate analysis will achieve accuracy levels closer to those of the AI deep learning model developed for this study. Our findings demonstrate the feasibility of AI-assisted imaging analysis in a clinical setting. In conclusion, the AI model outperformed the traditional statistical model in this dataset, but both approaches have potential value. Future studies with larger, multi-institutional datasets and robust validation are needed to confirm these results. We intend to apply and test our model on independent external datasets in future research to further assess its generalizability. Ultimately, quantitative imaging tools such as these could become valuable “objective” aids for radiologists and oncologists in evaluating pulmonary nodules and guiding patient management.

## Figures and Tables

**Table 1:** Demographic and clinical characterization of the study population (*n* = 47).

Characteristic	Frequency (*n*)	Percentage (%)
Age (years)	median, 70	[range 43–82]
Gender		
Male	17	36.17
Female	30	63.83
Male/female ratio	0.57	
Smoking		
Never	27	57
Past	9	20
Active	11	23
Pathological diagnosis		
Benign	15	32
Adenocarcinoma	25	53
Adenocarcinoma in situ	7	15
Tumor Staging ^1^		
0	22	47
IA1	22	47
IA2	1	2
IB	2	4
Overall lesion size on CT ^2^ (mm)	median 14	[range 8–32]
PT ^2^ malignant lesion size (mm)	median 10	[range 5–25]
Invasive part size (mm)	median 5	[range 0–20]

^1^ Tumor staging was determined according to the tumor invasive component [8]. Stage 0—An early stage lung cancer that is only in the top lining of the lung or bronchus and has not spread or invasiveness. Stage IA1—The tumor’s invasive part is smaller than 1 cm. Stage IA2—The tumor is only in the lung and is bigger than 1 cm but less than 2 cm. Stage IB—The tumor is larger than 3 cm but not larger than 4 cm. ^2^ Abbreviations: CT, computed tomography; PT, pathological assessment staging.

**Table 2:** Texture analysis of GGO lesions (*n* = 47) using MaZda software.

Variables	Benign (*n* = 15)	Malignant (*n* = 32)	*p*-Value *
S(0,1)Correlat	0.9 ± 0.03	0.93 ± 0.03	0.008
S(0,2)Correlat	0.68 ± 0.1	0.77 ± 0.11	0.012
S(0,3)Correlat	0.45 ± 0.17	0.58 ± 0.18	0.019
S(0,4)Correlat	0.25 ± 0.23	0.41 ± 0.23	0.027
S(1,-1)AngScMom	0.82 ± 0.07	0.86 ± 0.06	0.037
WavEnLH_s-2	72.63 ± 55.06	40.87 ± 17.6	0.045
S(0,5)Correlat	0.1 ± 0.28	0.27 ± 0.26	0.047
S(5,5)Entropy	1.96 ± 0.27	2.1 ± 0.21	0.054
WavEnLH_s-1	18.16 ± 12.9	11.3 ± 5.97	0.066
S(5,0)Entropy	2 ± 0.22	2.13 ± 0.21	0.068
WavEnHH_s-3	18.49 ± 14.32	12.09 ± 9	0.068
S(4,4)AngScMom	0.01 ± 0.01	0.01 ± 0.01	0.069
S(4,4)Entropy	2 ± 0.23	2.12 ± 0.21	0.071
S(2,-2)Correlat	0.51 ± 0.17	0.6 ± 0.16	0.072
S(5,-5)Entropy	1.97 ± 0.21	2.09 ± 0.21	0.078
S(3,3)Entropy	2.01 ± 0.23	2.13 ± 0.2	0.081
S(4,-4)Entropy	2.01 ± 0.21	2.12 ± 0.21	0.086
S(5,-5)AngScMom	0.01 ± 0.01	0.01 ± 0.01	0.096
S(5,5)AngScMom	0.02 ± 0.01	0.01 ± 0.01	0.098

* Values presented as mean ± standard deviation. *p*-values calculated using an unpaired *t*-test. All texture parameters are dimensionless units.

**Table 3:** Multivariate analysis of the logistic regression model.

Variable	Beta (Slope)	SE ^1^	Wald Test	*p*-Value
S(4,4)AngScMom	−149.0960	59.614000	6.255	0.012
WavEnLH_s-2	−0.0400	0.015000	7.119	0.008
Constant (intercept)	4.6930			

^1^ Abbreviation: SE, standard error.

**Table 4:** Sensitivity and specificity.

Outcome	Actual Positive Case	Actual Negative Case
Positive Test Result	True Positive	False Positive
Negative Test Result	False Negative	True Negative
	Sensitivity = TP ^1^/(TP + FN ^1^)	Specificity = TN ^1^/(FP ^1^ + TN)

^1^ Abbreviations: TP, true positive; FP, false positive; TN, true negative; FN, false negative.

## Data Availability

The datasets supporting the conclusions of this article are available upon reasonable request of the corresponding author.

## Abbreviations

The following abbreviations are used in this manuscript:

GGO	Ground-Glass Opacity
CT	Computed Tomography
AI	Artificial Intelligence
IARD	International Agency for Research on Cancer
COPD	Chronic Obstructive Pulmonary Disease
NSCLC	Non-Small Cell Lung Cancer
SCLC	Small Cell Lung Cancer
PET	Positron Emission Tomography
LDCT	Low-Dose Computed Tomography
CTR	Consolidation to Tumor Ratio
GGN	Ground-Glass Nodules
CMC	Carmel Medical Center
AUC	Area Under the Curve
ROC	Receiver Operating Characteristic
DS	Discrimination Score
PT	Pathological Assessment
SE	Standard Error
TP/TPR	True Positive/True Positive rate
FP/FPR	False Positive/False Positive Rate
TN	True Negative
FN	False Negative
